# Effect of addition of Nano hydroxyapatite particles on wear of resin modified glass ionomer by tooth brushing simulation

**DOI:** 10.4317/jced.53455

**Published:** 2017-03-01

**Authors:** Kiana Poorzandpoush, Ladan-Ranjbar Omrani, Shiva H. Jafarnia, Parisa Golkar, Mohammad Atai

**Affiliations:** 1 DMD, Pediatric Dentistry post graduate student, Dental Students Scientific research Center, Dental School, Tehran University OF Medical Sciences, Tehran, Iran; 2 MSc’s, Assistant Professor, Head of Dental Students Research Center, Operative Dentistry Department, Dental School, Tehran University of Medical Sciences, Tehran, Iran; 3 DMD, Tehran. Iran; 4 MSc’s, Operative Dentist; 5 Professor Iran Polymer and Petrochemical Institute. Tehran. Iran

## Abstract

**Background:**

Recently, incorporation of nanohydroxyapatite (NHA) has been suggested to improve the mechanical properties of glass ionomers (GIs). This study aimed to assess the effect of addition of NHA on wear of resin modified glass ionomer (RMGI) by tooth brushing simulation.

**Material and Methods:**

In this *in vitro*, experimental study, NHA in 1, 2, 5, 7 and 10wt% concentrations was added to Fuji II LC RMGI powder, and 48 samples (5×5mm) in five experimental and one control group (n=8) were fabricated. After polishing, cleaning and incubation at 37°C for three weeks, the samples were weighed and subjected to tooth brushing simulation in a toothpaste slurry according to ISO14569-1. Then, they were weighed again and the weight loss was calculated. The data were analyzed using one-way ANOVA and Tukey’s test.

**Results:**

The highest and the lowest weight loss was found in the 0% NHA (-1.052±0.176) and 5% NHA (-0.370±0.143) groups, respectively. Wear was significantly higher in 0% NHA group (*P*<0.05). No difference was detected in wear between 2 and 5wt% NHA or among 1, 7 and 10wt% NHA groups. Significant differences were noted in wear between 2 and 5wt% NHA and 1, 7 and 10wt% NHA groups (*P*<0.001).

**Conclusions:**

Incorporation of up to 10wt% of NHA increases the wear resistance of Fuji II LC RMGI. This increase was the highest when 2 and 5wt% NHA were added.

** Key words:**Glass ionomer, hydroxyapatites, nanoparticles, dental restoration wear.

## Introduction

Glass ionomer is a tooth-colored restorative material with extensive applications in dentistry (1) Success and longevity of tooth-colored restorations generally depend on the quality of the seal of cavity walls by the bonded restorative material (2). Glass ionomers are capable of forming a chemical bond to tooth structure (3). Moreover, they have favorable properties such as bio-compatibility, fluoride release, thermal expansion near to the tooth structure, radiopacity, preventing demineralization, and enhancing remineralization. However, they have some limitations as well including brittleness, low mechanical properties, long-term setting time, rough surface texture, low fracture toughness and low wear resistance, which limit their clinical use (4,5). Low wear resistance of these restorative materials is due to their inadequate strength immediately after application and their sensitivity to acids (6,7). To overcome the limitations of the conventional GIs (GIs), RMGIs were introduced, which have easier application, longer working time and higher initial strength. Addition of resin to GI in these systems resulted in higher flexibility and higher fracture toughness compared to the conventional GIs (8). The RMGIs easily bond to resin composite; however, they have free monomers, which may alter their biocompatibility compared to that of GIs (9).

The quality of RMGIs has greatly improved in the recent years; though, their durability as a restorative material in the oral environment depends on their mechanical and chemical properties (10). Low wear resistance is one drawback of GIs. These restorations undergo wear due to direct contact of teeth and restorations during mastication, oral parafunctional habits, tooth brushing with abrasive tooth pastes and chemical effects of nutritional factors (11). Wear due to tooth brushing can affect all dental surfaces and restorations and compromises their esthetics, increases surface roughness, which enhances plaque accumulation and subsequent soft tissue inflammation (8,12-16). Thus, wear resistance is an important quality for restorative materials especially for the restoration of posterior teeth (17).

Nanotechnology is among the recently employed techniques to improve the properties of materials without compromising esthetics. Incorporation of nanoparticles may improve the clinical efficacy of restorative materials in long-term (14). Needle-shaped NHA particles are added to restorative materials since they have optimal biological properties (18). They provide higher surface area due to smaller size of particles and have higher surface charge, which improves the strength and facilitates the use of glass ionomers (19,20).

This study aimed to assess the effect of incorporation of different weight percentages of NHA on the wear resistance and surface roughness of Fuji II LC RMGI. The null hypothesis was that addition of different weight percentages of NHA would have no effect on wear resistance of Fuji II LC RMGI.

## Material and Methods

This *in vitro*, experimental study was conducted on 48 RMGI samples. The samples were divided into six groups of eight including one control group without NHA and five experimental groups with 1, 2, 5, 7 and 10wt% NHA.

The NHA powder was weighed by a digital scale and added to Fuji II LC (GC Corporation, Tokyo, Japan) powder to reach 1, 2, 5, 7 and 10wt% concentrations of NHA. Based on a pilot study, the amount of powder required to fabricate eight samples with 1wt% of NHA in each experimental group was found to be 5g. The amount of nanoparticles required to obtain 5g powder was calculated to be 0.05g. Thus, to obtain samples with 1wt% NHA, 0.05g of NHA powder and 4.95g of RMGI powder were mixed. The values in other groups were also calculated as such.

The powders were gently mixed using a mortar and pestle for 20 minutes to obtain a homogenous powder. The powder was mixed with the liquid in 1:3.2 ratio according to the manufacturer’s instructions on a glass slab using a metal spatula in less than 25 seconds at room temperature. A 5×5mm mold was then placed on a glass slab, and the paste was placed in a plexiglass, in a 1 mm layer. Each layer was condensed, and light cured using an LED light-curing unit (Blue Phase, Ivoclar Vivadent, Schaan, Liechtenstein) with a light intensity of 600 mW/cm2 for 40 seconds. After insertion of last layer, another glass slab was placed over the mold and condensed excess RMGI leaked out a smooth surface obtained, the last layer was cured, and the samples were removed from the mold and light cured from all four sides for 40 seconds. The output of the light-curing unit was checked by a radiometer.

Defective samples were excluded and replaced with new ones. After removing the samples from the molds, they were polished with 1200 grit abrasive paper under irrigation with distilled water. They were placed in an ultrasonic bath. Samples were coded and incubated at 37°C in tap water. Each sample was weighed every 48 hours for three weeks until achieving a constant weight in five consecutive measurements. The weight was measured by a digital scale (Mettler, USA) with 0.0001g accuracy.

-Wear test:

Wear test of samples was performed according to ISO 14569-1(21) using a tooth brushing simulator (V8 cross brushing machine, Oj Andishan Spadan, Iran, Tehran). This machine has eight steel retainers and eight acrylic resin stubs to hold one sample on each stub. The heads of Aqua Fresh toothbrushes with 50 medium nylon bristles in 20 tufts with 400g load were used vertically on the lubricated surfaces for a total of 50,000 strokes, corresponding to five years of tooth brushing in the clinical setting. The frequency was adjusted to 100 strokes/minute (complete back and forth motion).

A slurry was prepared by mixing 20g of toothpaste (Crest Complete 7) and 40mL of distilled water. The concentration was the same for all samples. The samples were immersed in the slurry in such a way that their entire surfaces were soaked in the slurry. The slurry was prepared right before the experiment and constantly stirred to prevent precipitation of abrasive particles. Wear test was performed at room temperature (23°C±1). The toothbrushes were changed for each new cycle of 50,000 (20).

After completion of testing, the samples were removed from the mold and rinsed under running water. Then, they were placed in an ultrasonic bath (StarSonic 35, 120 w, Italy) for 10 minutes. Each sample was weighed and the weight loss for each sample was separately calculated. The change in weight was calculated by subtracting the final weight from the primary weight. Paired t-test was applied for statistical analysis of weight loss and its comparison between the groups.

-Statistical analysis

SPSS software was used for data analysis. One-way ANOVA was applied to determine the effect of weight percentage of added NHA on the wear (weight loss) of samples. In case of presence of a significant difference, post-hoc Tukey’s test was applied for pairwise comparison of groups.

## Results

**Figure 1:**
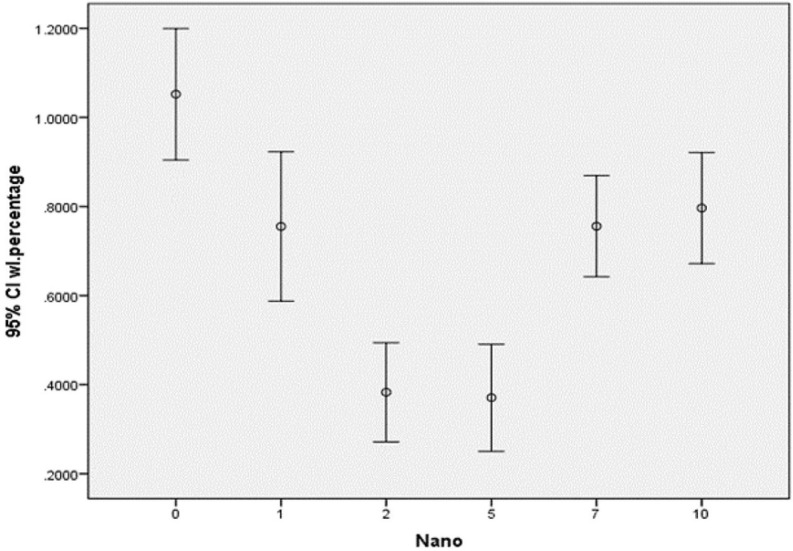
The mean and 95% confidence interval of weight loss in the six groups.

The highest mean weight loss occurred in 0% NHA group (-1.052%±0.176) and the lowest mean weight loss occurred in 5wt% group (-0.370±0.143). The mean and standard deviation of weight loss in the six groups are shown in  Table 1. The mean weight loss with 95% confidence interval in the six groups is shown in figure 1. According to one-way ANOVA, significant differences existed in weight loss following tooth brushing among the groups (*P*<0.001). Thus, post-hoc Tukey’s test was applied for pairwise comparisons and showed that the control group (0wt% of NHA) had the highest wear with significant differences with other experimental groups (*P*<0.05). The groups with 2wt% and 5wt% NHA showed the least wear compared to other groups. No significant differences were noted in 2wt% and 5wt% concentrations of NHA in terms of wear (*P*>0.05). No significant differences were found in 1, 7 and 10wt% concentrations of NHA in terms of wear (*P*>0.05). Significant differences were noted in wear between 2 and 5wt% groups with 1, 7 and 10wt% groups in terms of wear (*P*<0.001).

**Table 1:**
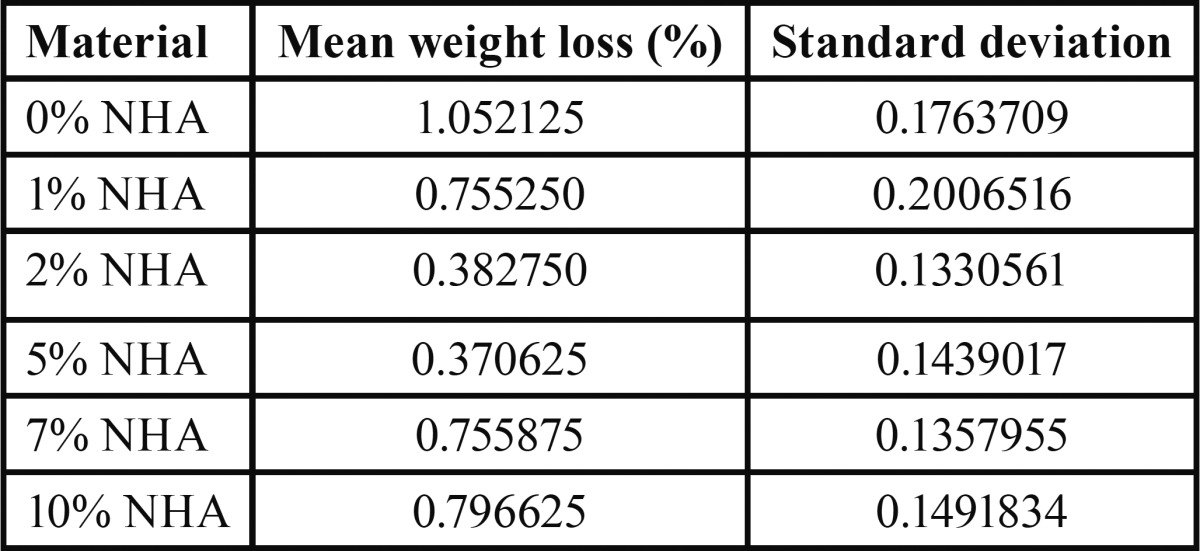
The mean and standard deviation of weight loss in the six groups.

## Discussion

The RMGIs are used in patients with high risk of caries due to their fluoride release potential and chemical and micromechanical bond to tooth structure (21). Glass ionomers have extensive applications as luting cement, filling material and liner. However, poor mechanical properties limit their application in high-stress bearing occlusal areas (11,19). To improve the physical properties of glass ionomers, several mechanisms have been suggested to increase their cross-linking and subsequently physical properties (19).

Wear resistance is among the most challenging properties of direct restorative materials (22). If not controlled, wear can impair the masticatory function (23). Wear due to tooth brushing may occur at any surface but has a higher frequency in the buccal surfaces of teeth (24). Surface roughness is affected by tooth brushing and abrasive agents used. A smooth surface not only provides acceptable esthetics, but also prevents accumulation of biofilm and subsequent discoloration, plaque retention and inflammation (13-15).

Attempts have been made to improve the mechanical properties of GIs by addition of fibers, metal particles and nano-size fillers. Addition of NHA particles to GIs has also been suggested since NHA particles have a crystalline structure similar to tooth apatite and have shown to be able to enhance remineralization and decrease microleakage of restorations (22,24). It seems that hydroxyapatite nanobioceramics dissolve in acidic solutions. Thus, after mixing the RMGI powder with the poly acid liquid, calcium ions are released from the surface of NHA particles, which increase acid-base and cross-linking reactions within the GI structure and further reinforce it (25).

Our results showed that addition of NHA by up to 10wt% to Fuji II LC increased its wear resistance. Increase in wear resistance was higher in 2 and 5wt% NHA groups compared to the control and 1, 7 and 10wt% groups (*P*<0.001). These findings indicate the strong interaction of GI matrix with NHA particles.

Mohammadi Basir *et al.*, in their electron microscopic study in 2013 showed homogenous distribution of nanoparticles in the matrix in presence of 5wt% NHA. Combination of glass particles, which are relatively large in size and NHA particles with smaller dimensions results in extensive distribution of particles and increases their packing density and improves their mechanical property (26).

In the current study, addition of NHA by more than 5wt% resulted in greater weight loss in samples (but lower than that of the control group). This finding may be due to several reasons: In weight percentages over 5% of NHA, agglomerates form within the glass ionomer resin matrix, which serve as defect sites and can decrease the wear resistance. Garoushi *et al.*, in 2011 evaluated the effect of addition of 0, 10, 15, 20 and 30wt% of silica nanoparticles to resin matrix of microfilled composites and found that addition of nanoparticles by up to 30wt% did not improve the wear resistance of microfilled composites; their findings were in contrast to ours, which is probably due to the fact that they evaluated microfilled composites while we evaluated RMGIs. They showed the formation of agglomerates in presence of over 10wt% of nanoparticles, which interfered with light penetration and polymerization of composite and even resulted in light diffraction (27). Thus, it seems that in higher than 5wt% concentration of nanoparticles, polymerization of resin component of RMGIs may not occur completely and this incomplete polymerization can adversely affects its physical and mechanical properties. In 7 and 10wt% concentrations of NHA, decreased wear resistance may be due to incomplete penetration of light and subsequently inadequate polymerization. Filler size, quality of bond between filler and matrix and degree of polymerization of resin matrix can all affect the wear resistance of set material (28).

In 7 and 10wt% NHA, due to smaller size and higher surface area of nanoparticles compared to glass particles, the liquid seems not to be sufficient for maintaining the NHA particles and thus, increasing the weight percentage of NHA results in loose adhesion between the particles and ionomer matrix and interferes with the acid-base reaction. Another possible explanation is that glass particles serve as reinforcing fillers and in higher weight percentages of NHA particles, the percentage of glass particles decreases and thus, its wear resistance decreases as well. Also, in presence of higher than 5wt% NHA, its viscosity increases. Thus, the process of powder and liquid mixing becomes more difficult and consequently, non-homogenous samples may form.

Weir et al., in 2012 reported that following the addition of 0, 10, 20 and 30wt% nano-silica particles, wear of nanocomposites was the same as that of control samples (29). This result may be due to the formation of agglomerates and impaired curing of composites, resulting in increased wear of nanocomposites.

In our study, the most efficient distribution of particles in the matrix was seen in 5wt% group. It seems that by increasing the percentage of NHA, fillers no longer have adequate coverage by the resin matrix and thus, following tooth brushing, unreacted nanoparticles are detached from the surface, leaving small, nano-sized pits on the surface. In contrast in 5wt% and lower concentrations of NHA, lower percentage of unreacted nanoparticles is present and thus, by simulated tooth brushing, the odds of detachment of glass particles are higher.

This study had an *in vitro* design and suffered the limitations of *in vitro* studies; thus, generalizability of results to the clinical setting must be done with caution. Future studies are required to assess the effect of addition of 2 and 5wt% NHA on the physical and mechanical properties as well as the biocompatibility of RMGIs.

Within the limitations of this study, addition of specific weight percentages of NHA up to 10wt% increased the wear resistance of Fuji II LC RMGI; this increase was the highest in 2 and 5wt% groups. Thus, addition of 2 and 5wt% of NHA to RMGI not only improves its biological properties, but also increases its wear resistance.
